# Drug misuse, tobacco smoking, alcohol and other social determinants of tuberculosis in UK-born adults in England: a community-based case-control study

**DOI:** 10.1038/s41598-020-62667-8

**Published:** 2020-03-27

**Authors:** Patrick Nguipdop-Djomo, Laura C. Rodrigues, Peter G. Smith, Ibrahim Abubakar, Punam Mangtani

**Affiliations:** 1 Department of Infectious Disease Epidemiology, Faculty of Epidemiology and Population Health, London School of Hygiene & Tropical Medicine, London, UK; 2 Institute of Epidemiology and Health, and Centre for Infectious Disease Epidemiology, Faculty of Population Health Sciences, University College London, London, UK

**Keywords:** Tuberculosis, Lifestyle modification, Preventive medicine, Epidemiology, Risk factors

## Abstract

Addressing social determinants of tuberculosis (TB) is essential to achieve elimination, including in low-incidence settings. We measured the association between socio-economic status and intermediate social determinants of health (SDHs, including drug misuse, tobacco smoking and alcohol), and TB, taking into account their clustering in individuals. We conducted a case-control study in 23–38 years old UK-born White adults with first tuberculosis episode, and randomly selected age and sex frequency-matched community controls. Data was collected on education, household overcrowding, tobacco smoking, alcohol and drugs use, and history of homelessness and prison. Analyses were done using logistic regression models, informed by a formal theoretical causal framework (Directed Acyclic Graph). 681 TB cases and 1183 controls were recruited. Tuberculosis odds were four times higher in subjects with education below GCSE O-levels, compared to higher education (OR = 3.94; 95%CI: 2.74, 5.67), after adjusting for other TB risk factors (age, sex, BCG-vaccination and stays ≥3 months in Africa/Asia). When simultaneously accounting for respective SDHs, higher tuberculosis risk was independently associated with tobacco smoking, drugs use (especially injectable drugs OR = 5.67; 95%CI: 2.68, 11.98), homelessness and area-level deprivation. Population Attributable Fraction estimates suggested that tobacco and class-A drug use were, respectively, responsible for 18% and 15% of TB cases in this group. Our findings suggest that socio-economic deprivation remains a driver of tuberculosis in England, including through drugs misuse, tobacco smoking, and homelessness. These findings further support the integration of health and social services in high-risk young adults to improve TB control efforts.

## Introduction

Tuberculosis (TB) is strongly related to social exclusion and inequality^1^. Although most TB cases in high-income countries are in migrants from high incidence countries, cases in the native-born population are often concentrated in vulnerable deprived and marginalised groups^2^. Poverty, lower education, reduced access to good healthcare, and poor living standards increase the risk of TB as well as deprivation-related behaviours harmful to health^3^, such as alcohol and controlled-drug misuse^4^ and tobacco smoking^5^. However, TB control strategies have often emphasised medical technologies (including preventive treatment and vaccination, early and better diagnosis, effective treatment, and management of co-morbidities), with less focus on tailoring strategies to the socioeconomic circumstances conducive of higher disease burden^6^.

The World Health Organization (WHO) 2015 strategy to eliminate TB by 2035 recognises this target cannot be achieved without also prioritising the social determinants of TB^7^, including in low-TB incidence countries, which are closer to TB elimination^8^. The pathways through which poverty affects TB risk have however received limited attention in these settings. Most studies have been ecological, comparing rates in geographic areas with different socio-economic levels^9^. Most individual-level studies in the setting have focused on either contrasting patients’ characteristics by groups (e.g. native vs foreign born, age groups, and drug sensitive vs drug resistant), or comparing the association between these social risk factors and treatment outcome, rather than disease risk^2^.

In England, social deprivation appears to contribute significantly to TB risk. Over 20% of notified UK-born TB cases report at least one of homelessness, drug misuse, alcohol abuse, or history of prison stay, compared to 10% in foreign-born cases^10^. Subjects with such social risk factors are younger (about 60% aged 15–44 years), more likely to be from White ethnic background, and present more commonly with pulmonary TB, the most communicable disease form^10^. Recurrent TB, drug-resistance and poor treatment outcomes are also more frequent in this group^10^. However, these social risk factors tend to cluster in individuals, therefore providing an additional challenge to disentangling their respective effects. A better understanding of the relative contribution of these social determinants of health to the risk of TB in the native White population could help tailor TB control efforts^9^.

We report on a case-control study investigating the associations between individual socio-economic status (SES) and a range of social determinants of health (including drug misuse, tobacco smoking, alcohol and homelessness), and the risk of TB in UK-born young adults of White ethnic background.

## Methods

### Study design, setting and participants

We used data from a community-based case-control study conducted between 2012 and 2014 across England measuring the duration of school-aged BCG (Bacillus Calmette-Guerin vaccine) protection against tuberculosis in England^11^.

**Figure 1:**
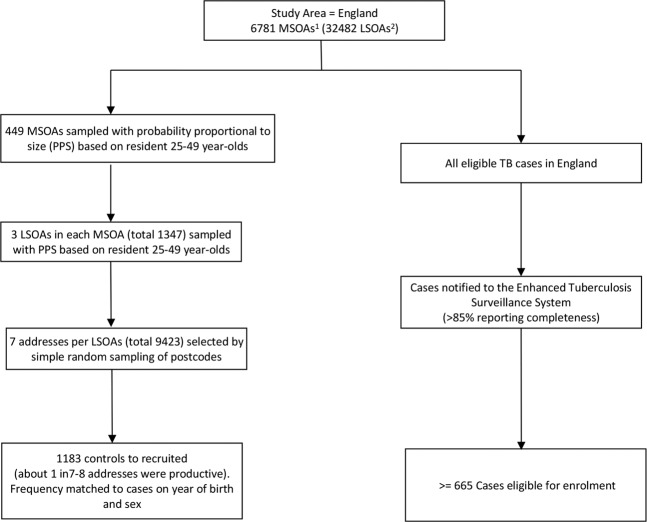
Summary of sampling strategy for cases and controls across England. ^1^MSOA = Mid-level Super-Output areas; contiguous LSOAs, constrained by the 2003 English local authority boundaries, average population 7,200 inhabitants. ^2^LSOA = Lower level Super-Output Areas (LSOAs); designed by the Office for National Statistics to have a socially homogeneous population with an average 1500 residents in each area. The census Output Areas are the smallest statistical enumeration level in England, and the Super Output Areas are the smallest grouping of contiguous output areas used to generate official statistics.

Cases were subjects with a first episode of TB diagnosed at age 23 to 38 years between 2003 and 2012 notified to the UK Enhanced Tuberculosis Surveillance system (ETS). Controls were those never diagnosed with TB, frequency-matched to cases on sex and birth-year cohort (±2 years), with a ratio of approximately two controls per case. Controls were randomly sampled from the same communities as cases based on cluster-sampling with probability proportional to the population size of small areas, then simple random sampling of residential addresses from the UK Postcode Address File (Fig. 1). Cases with known HIV infection and those living in state institutions (e.g. prisons) were excluded. Data on social factors were collected as part of a study to measure the durability of BCG vaccine effectiveness 10–30 years post school aged-vaccination in the UK (median age of 13 years), hence the restriction to the 23–38 years age-group; details are reported elsewhere^11^.

### Ethics

Ethical approval was obtained from the UK National Health Services Research Ethics Committee (NHS-REC 11/H1102/11), and the London School of Hygiene and Tropical Medicine (LSHTM) Research Ethics Committee (Approval 5996). All participants provided written informed consent. All study methods were performed in accordance with the relevant guidelines and regulations.

### Variables

Information was collected by interview on socio-demographic characteristics, a number of lifestyle variables and other TB risk factors. These included age, sex, residential postcode, and highest educational level attained. Residential postcodes linked to the Office for National Statistics data allowed their categorisation into quintiles of small-area indices of multiple deprivation (IMD).

Other potential TB risk factors included history of ≥12 weeks homelessness, prison stay (in the UK or abroad), stays of ≥3 months duration in high TB incidence regions (Africa and Asia), and BCG vaccination. To minimise information bias, subjects self-entered and electronically locked tobacco, alcohol and drug use, history of homelessness, and prison stay information on computer-assisted software, thus inaccessible to the interviewer.

### Statistical analysis

Socio-economic variables were grouped into distal (educational level, residence based on area-level deprivation and household overcrowding) and intermediate social factors (tobacco smoking, alcohol drinking, drug misuse, history of homelessness and prison stay). We posited a causal framework of the relationship between these variables and the TB risk, as well as to each other, based on background knowledge represented in a directed acyclic graph (DAG), to inform the statistical models. The matching variables (birth cohort, sex) and other TB determinants (BCG vaccination and long stays in high TB-incidence) were considered a-priori confounders and included in all adjusted models.

We assumed that educational level affects employment opportunities and income in later life, and through these, area-level deprivation at the place of residence and household overcrowding level. The educational level and socio-economic status may also affect risk of tuberculosis via other pathways not measured in this study. However, we assumed that education level has a dual effect on the intermediate social determinants, by itself and via its effect on socio-economic status (Supplementary Fig. 1). This assumption allows control for indirect paths between the intermediate variables and TB that do not include education level, which could otherwise have been missed.

The distribution of variables was compared in cases and controls. Characteristics of study participants with data missing for at least one variable were compared to those with complete data. Correlations between related variables were also examined, notably between lifestyle variables (use of tobacco, alcohol and controlled drug), and between history of prison and homelessness.

Two groups of logistic regression models were built using Stata 14, respectively measuring the overall association between education level, then that of each intermediate factor in the DAG, and TB. Baseline models (controlling for birth-cohort and sex) and adjusted models (controlling for other confounders and appropriate variables from the DAG) were fitted. For the overall association between educational level the fully-adjusted logistic regression model controlled for birth-cohort, age and sex, BCG status and stays in high TB regions. For the separate investigation of other SDHs and TB risk, the fully adjusted model (based on the DAG) included all a-priori confounders, education level, as well as all other variables in the DAG. The variables Class B/C drug (e.g. Cannabis, Amphetamines, Benzodiazepines, Qat, Glue, Solvents, Speed or other amphetamines etc.) misuse and Class A drug (e.g. Ecstasy, Cocaine, Crack Cocaine, Heroin, LSD, Psychedelics (e.g. “magic” mushrooms)), misuse were strongly correlated, preventing their simultaneous inclusion in the model. These were therefore measured in turn in separate models.

The final models were based on observations with complete data for all variables. Significance testing and tests for trend for ordered categorical variables in which level-specific estimates suggested a trend were done using likelihood ratio tests.

We conducted sensitivity analyses using multiple imputation by chained equations (MICE) to impute missing data. MICE models included all variables in the fully-adjusted model, and case/ control status. Twenty datasets were imputed, repeating the complete-case regression models and estimates combined using Rubin’s rules.

For key modifiable risk factors, we estimated the population attributable fraction (PAF) for our study’s target population, using the formula **PAF** = **∑p**_**i**_**(aOR**_**i**_ **− 1)/aOR**_**i**_, where p_i_ is the proportion of cases with exposure level i for a specific risk factor, and aOR_i_ is the adjusted odds ratio for level i of that risk factor^12^.

## Results

### Overview of study sample and characteristics

There was 9% more female control than cases, but comparable distributions by birth cohorts (Table 1). Complete data were available for 88% (1618/1864) of participants, with missing data mostly seen for alcohol drinking (3%), tobacco smoking (3%) and education level (3%). Over 40% of cases lived in the most deprived quintile of residence, and 20% reported less than 13 years of formal education compared to 5% of controls. About 25% of cases smoked daily with lifetime consumption >10 pack-years, and 18% reported alcohol drinking above 14 units/week, compared with 13% and 11% respectively in controls. Ten percent of cases reported using injectable class A drugs compared to 1% of controls. Using class B/C drugs was strongly correlated with using class A drugs (Spearman correlation coefficient = 0.76). Social risk factors clustering was more common in cases than controls (e.g. 15% of cases reported at least two of class A drug use, history of homelessness or prison stay, compared to 3% of controls – Supplementary Fig. 2).

**Table 1:** Characteristics of Study Participants, England.

Variable	Cases (%) (n = 681)	Controls (%) (n = 1183)
**Sex**		
Female	345 (51%)	710 (60%)
Male	336 (49%)	473 (40%)
**Birth cohort**		
1965–1969	65 (10%)	175 (15%)
1970–1974	179 (26%)	318 (27%)
1975–1979	216 (32%)	263 (22%)
1980–1984	152 (22%)	264 (22%)
1985–1989	69 (10%)	163 (14%)
**Education level**^**a**^		
Less than O level, GCE or GCSE	132 (19%)	75 (6%)
O level, GCE or GCSE	208 (31%)	366 (31%)
A level, SCE Higher (Scotland)	91 (13%)	250 (21%)
Degree or Teaching degree	218 (32%)	461 (39%)
Missing	31 (5%)	31 (3%)
**BCG vaccination status**		
BCG vaccinated	509 (75%)	1024 (87%)
Unvaccinated	164 (24%)	154 (13%)
Missing	8 (1%)	5 (0·4%)
**Stay of 3 months or more in High TB-incidence areas (Africa or Asia)**		
No	610 (90%)	1126 (95%)
Yes	71 (10%)	57 (5%)
**Small area-level deprivation**		
Least deprived quintile	64 (9%)	238 (20%)
2^nd^ quintile	101 (15%)	236 (20%)
3^rd^ quintile	107 (16%)	237 (20%)
4^th^ quintile	131 (19%)	236 (20%)
Most deprived quintile	278 (41%)	236 (20%)
**Persons per bedroom**		
<2 persons	587 (86%)	1104 (93%)
≥2 persons	77 (11%)	77 (7%)
Missing	17 (3%)	2 (0.2%)
**Tobacco smoking**		
Never smoked	191 (28%)	508 (43%)
Past smoker	94 (14%)	215 (18%)
Occasional/Daily <10 pack-years	202 (30%)	277 (23%)
Daily 10–19.9 pack-years	119 (17%)	106 (9%)
Daily ≥20 pack-years	57 (8%)	47 (4%)
Missing	18 (3%)	30 (3%)
**Typical Alcohol consumption**		
None	36 (5%)	49 (4%)
Up to 40 g (5 units)/week	324 (48%)	658 (56%)
41–112 g (5–14 units)/week	172 (25%)	300 (25%)
>112 g (>14 units)/week	120 (18%)	130 (11%)
Missing	29 (4%)	46 (4%)
**Class B and C drugs misuse**		
Never used	378 (56%)	818 (69%)
Last used >10 years ago	63 (9%)	135 (11%)
Last used 1–10 years ago	95 (14%)	106 (9%)
Used in last year	130 (19%)	93 (8%)
Missing	15 (2%)	31 (3%)
**Class A drugs misuse**		
Never used	426 (63%)	921 (78%)
Last used >10 years ago	44 (6%)	88 (7%)
Last used ≤10 years ago	128 (19%)	131 (11%)
Current user	66 (10%)	12 (1%)
Missing	17 (2%)	31 (3%)
**History of homelessness and sleeping rough**		
Never	556 (82%)	1104 (93%)
≤12 weeks	61 (9%)	43 (4%)
>12 weeks	57 (8%)	25 (2%)
Missing	7 (1%)	11 (1%)
**History of prison in UK and/or abroad**^**†**^		
Never in prison	593 (87%)	1132 (96%)
Ever in prison^b^	83 (12%)	35 (3%)
Missing	5 (1%)	16 (1%)

^a^In terms of years of formal education: Degree level [~17 years, starting about age 5 years], General Certificate of Education (GCE) A-levels or equivalent level [~14 to 17 years], GCE O-levels, General Certificate of Secondary Education (GCSE) or equivalent [~12 to 13 years], and below O-levels [<12 years].

^b^including 10 (1.5%) cases and 2 (0.2%) controls with history of prison abroad.

### Social determinants and risk of tuberculosis

The odds of TB in subjects with education level <13 years duration was nearly four times higher than in those with degree-level education but without a clear gradient among those with 13 years of education of more. The associations were little changed after adjusting for age, sex, BCG vaccination status and long stays in high TB areas, and after multiple imputation of missing data (Table 2).

**Table 2:** Association Between Education Level and Tuberculosis in White UK-born Subjects Aged 23–38 Years, England.

Variable	Baseline model^a^			Adjusted model^b^		
	OR	95%CI	p-value	aOR	95%CI	p-value*
**Complete Case analysis (578 cases and 1060 controls)**						
Degree or Teaching degree	1			1		
A level, SCE Higher	0.78	0.58, 1.06		0·79	0.58, 1.08	
O level, GCE or GCSE	1.19	0.93, 1.53	<0·001	1.28	0.9, 1.66	<0.001
None	3.84	2.70, 5.47		3.94	2.74, 5.67	
**MICE**^**c**^ **Imputed datasets (681 cases and 1183 controls)**						
Degree or Teaching degree	1			1		
A level, SCE Higher	0.82	0.61, 1.10		0.85	0.63, 1.15	
O level, GCE or GCSE	1.30	1.02, 1.65	<0.001	1.41	1.10, 1.81	<0.001
None	4.21	2.92, 5.65		4.21	2.99, 5.92	

^a^Baseline model adjusted for age (birth cohort) and sex.

^b^Adjusted model: Educational level adjusted for age, sex, BCG vaccination status and stays ≥3 months in high TB incidence areas (Africa or Asia). Other variables not included in model because assumed to be mediators in causal framework.

^c^MICE = Multiple Imputation using Chained Equations.

^*^Likelihood Ratio Test P value of overall association.

A strong association between area-level deprivation and TB was seen in subjects living in the most deprived fourth and fifth quintiles areas, respectively, compared to those living in the least deprived quintile, after adjustment for confounding ([aOR=1.74; 95%CI = 1.16, 2.59] and [aOR=3.30; 95%CI = 2.23, 4.88] respectively) (Table 3).

**Table 3:** Association Between Intermediate Social Determinants of Health and Tuberculosis in White UK-born Subjects Aged 23–38 Years, England^a^.

Variable	Baseline model^b^ (578 cases and 1060 controls)			Fully adjusted Model^*c*^ (578 cases and 1060 controls)			Multiple imputation fully adjusted (681 cases and 1183 controls)		
	OR	95%CI	p-value	aOR	95%CI	p-value	aOR	95%CI	p-value
**Quintiles of Index of Multiple Deprivation**									
Least deprived quintile	1			1			1		
2nd quintile	1.70	1.16, 2.51		1.76	1.18, 2.64		1.64	1.12, 2.41	
3rd quintile	1.60	1.09, 2.35	<0·001	1.51	1.01, 2.27	<0.001	1.54	1.05, 2.26	<0.001
4th quintile	1.95	1.34, 2·85		1.74	1.16, 2·59		1.87	1.29, 2·73	
Most deprived quintile	4.38	3.07, 6.24		3.30	2.23, 4.88		3.30	2.29, 4.75	
**Persons per bedroom (ppb)**									
<2 ppb	1			1				1	
≥2 ppb	2.01	1.40, 2.88	<0.001	1.42	0.95, 2·12	0.091	1.34	0.92, 1.95	0.128
**Tobacco smoking**									
Never smoked	1			1			1		
Past smoker	1.33	0.98, 1.82		1.17	0.83, 1·65		1.01	0.73, 1.41	
Occasional/<10pk-yr	1.89	1.45, 2.46	<0·001	1.25	0.92, 1.69	0.008*	1.24	0.93, 1.65	0.004*
Daily 10 to 19·9 pk-yr	2.67	1.90, 3·75		1.61	1.09, 2·38		1.61	1.12, 2.32	
Daily ≥20 pk-yr	3.49	2.18, 5.59		1.72	0.98, 3.01		1.66	0.99, 2.76	
**Typical Alcohol drinking**									
Non-drinker/ ≤40 g/wk	1			1			1		
41–111 g/wk	0.98	0.77, 1·25	0.015	1.00	0.76, 1·3		1.10	0.86, 1.43	
≥112 g/wk	1.54	1.13, 2.10		1.06	0.75, 1.51	0.936	1.20	0.86, 1.67	0.494
**Class B/C drug misuse**									
Never	1			1			1		
>10 years ago	0.88	0.62, 1.25		0.73	0.49, 1.08	0.004	0.74	0.51, 1.06	0.004
1–10 years ago	1.94	1.40, 2.7	<0.001	1.55	1.07, 2.23		1.52	1.07, 2·16	
<1 year ago	2.78	2.00, 3.85		1.49	1.00, 2.20		1.43	0.99, 2·07	
**Class A drug misuse**									
Never	1			1			1		
>10 years ago	0.91	0.6, 1.38		0.72	0.45, 1.14		0.79	0.51, 1.22	
≤10 years ago	1.91	1.42, 2.57	<0·001	1.51	1.07, 2.12	<0.001	1.46	1.05, 2.02	<0.001
Injectable (ever)	10.57	5.44, 20.53		5.67	2.68, 11.98		5.36	2.63, 10.90	
**BCG vaccination status**									
No	1			1			1		
Yes	0.45	0.35, 0.57	<0.001	0.51	0.38, 0.68	<0.001	0.51	0.39, 0.67	<0.001
**Stay of 3 months or more in High TB incidence areas Africa or Asia**									
No	1			1			1		
Yes	2.27	1.54, 3.34	<0.001	2.67	1.74, 4.08	<0.001	2.63	1.76, 3.94	<0.001
**History of homelessness**									
Never	1			1			1		
≤12 weeks	2.92	1.86, 4.59	<0.001	1.66	0.99, 2.79	0.005*	1.51	0.94, 2.41	0.008*
>12 weeks	4.35	2.56, 7.40		2.01	1.11, 3.63		1.88	1.09, 3.23	
**History of prison stay**									
No	1			1			1		
Yes	3.88	2.49, 6.04	<0.001	1.34	0.79, 2·28	0.273	1.45	0.88, 2.38	0.144

^a^The associations between TB and the a priori confounders BCG vaccination and long stays in high TB incidence parts of the world are also reported in the table.

^b^Baseline model is controlling for frequency-matching variables birth cohort and sex.

^c^Fully adjusted model further controls for education level and all the variables presented in the table.

*p-value of test for trend.

The strongest risk factor among the intermediate social determinants was misuse of class A injectable drugs, with five times higher TB odds (aOR = 5.67; 95%CI = 2·68, 11.98) compared to those who never misused class A drugs. The TB risk was also 50% higher in those who reported using either non-injectable class A or class B/C drugs within the past 10 years. There was strong evidence (*P* = 0.008) of a dose-response association between level of tobacco smoking and TB, compared to never-smokers, but no clear association between alcohol drinking levels and TB. History of homelessness of up to 12 weeks and >12 weeks were respectively associated with 66% (aOR = 1.66; 95%CI = 0.99, 2.79) and double (aOR = 2.01; 95%CI = 1·11, 3.63) higher TB odds.

BCG vaccination on average halved the risk of TB in those vaccinated 10 to 30 years earlier (aOR = 0.51; 95%CI = 0.38, 0.68), and travel for 3 months or more in high-TB burden parts of the world was associated with more than a doubling in the risk of disease.

### Population attributable fractions

While the association between TB and tobacco smoking was not strong, avoiding smoking could reduce TB cases in our target population by 18% (Table 4) because of the high prevalence of smoking (16% controls smoked >10 pack-year daily - Table 1). Similarly, preventing Class-A drugs misuse could prevent 15% TB cases, 8% by stopping injectable Class-A drug abuse. BCG vaccination may have helped prevent about 12% cases and eliminating homelessness could reduce 7.6% of TB notifications. The respective impact of these risk factors is not assumed additive; the joint PAF for any combination of risk factors is not the sum of their respective PAFs.

**Table 4:** Estimates of Tuberculosis Population Attributable Fraction (PAF) for Specific Risk Factors in White UK-born Subjects Aged 23–38 Years, England.

Risk Factor	Number of Cases	% Cases (N = 578)	aOR	Level specific PAF	Total PAF^a^
**Tobacco smoking**					
Past smoker	91	16%	1.17	2.3%	
Occasional/<10pk-yr	173	30%	1.25	6.0%	18.0%
Daily 10 to 19·9 pk-yr	96	17%	1.61	6.4%	
Daily ≥20 pk-yr	47	8%	1.72	3.3%	
**Class A drug misuse**					
<10 yrs	110	19%	1.51	6.4%	14.7%
Injectable	57	10%	5.67	8.2%	
**Class B/C drug misuse**					
1–10 years ago	84	15%	1.55	5.3%	11.6%
<1 year ago	109	19%	1.49	6.2%	
**BCG**					
Unvaccinated	140	24%	1.96	11.8%	11.8%
**Stay of 3 months or more in High TB-incidence areas (Africa or Asia)**					
Yes	62	11%	2.67	6.9%	6.9%
**History of homelessness**					
≤12 weeks	50	9%	1.66	3.6%	7.6%
>12 weeks	46	8%	2.01	4.0%	

^a^**PAF** = **∑p**_**i**_**(aOR**_**i**_ **− 1)/aOR**_**i**_, where p_i_ is the proportion of cases with exposure level i for a specific risk factor, and aOR_i_ is the adjusted odds ratio for level i of that risk factor.

### Missing data and sensitivity analysis

The 226 subjects with data missing for at least one variable (12% overall, 15% cases and 10% controls, *P* = 0.003) were slightly less educated, more deprived, heavier tobacco smokers and alcohol drinkers and more likely to have a history of homelessness, compared to those 1638 (88%) with complete data (Supplementary Table 1). The results from statistical analyses after multiple imputation of missing data were similar to those of the complete case analyses (Table 3).

## Discussion

Declines in TB incidence among UK-born in England since 2012 varied by ethnicity. In the White ethnic group TB rates have remained relatively stable^10^. Social deprivation may explain in part these relatively stagnant rates, with TB rates in White UK-born in the most deprived areas more than 4 times higher than least deprived areas, and a higher proportion of reporting at least one social risk factor compared to other ethnic groups^10^. We have, to our knowledge, made the first attempt at a causal framework analysis of how the association between overall socio-economic status (SES) and TB in the native population from a high-income low-TB burden setting may be mediated by intermediate social factors. We found that amongst UK-born White young adults in England, the risk of TB was four times higher in those with less than 13 years education than those with A-level and above. This association may be explained by the link between low educational level and small-area level deprivation, tobacco smoking, misuse/abuse of controlled drugs, and homelessness, and in turn their effect on TB risk.

In low-incidence settings, there is data showing that poverty and lower SES are associated with greater risk of *Mycobacterium tuberculosis* infection^9^, as well as delay to diagnosis and treatment^13^, and poorer treatment outcome^14^; but there is limited data measuring the association of individual SES to the risk of active TB. The direct comparison of our findings to other studies is complicated by variations in individual SES measures used across settings. We used highest educational attainment, which is assessed to be a good indicator of SES, because it is highly correlated with parental SES, as well as a strong predictor of future employment and earnings, marking both early life influences and adult SES^15^. Our findings that the risk of TB is nearly four-fold higher in individuals with lower education levels is consistent with the few published studies that have measured the association between individual-level SES and TB. In their case-control study of risk factors of TB in adults in Washington in 1988–90, Buskin *et al*. used family income, years of education and housing conditions to create a composite binary SES variable^16^; they found that the risk of TB in the lowest SES category was about four times higher than that of the highest SES group. A case-control study in Greenland, in 2004–06, using occupation as the proxy-measure for SES, noted that TB risk four times greater in those unemployed than in those in work or in studies^17^.

The association between tobacco smoking and higher TB risk is consistent with results from two UK case-control studies in which current smokers were found to be on average 60% more likely to develop TB than non-smokers^18^. Similar results were also reported from a USA study^19^, and systematic reviews^20^. It is not surprising that lower SES increases the risk of TB through tobacco smoking. Studies have consistently found higher prevalence of tobacco smoking and younger age at smoking initiation in subjects from lower SES^5,21^. Tobacco-related loss of mucosal immunity in the respiratory tract may increase the risk of *TB* infection in smokers; and impair both innate and adaptive immune responses leading to higher risk of progression to disease^22,23^. The dose-effect relationship between smoking and TB risk in our study further supports a direct effect of tobacco smoking on TB risk.

Subjects who admitted to using either of class C, B or non-injectable class A drugs in the past 10 years were more likely to develop TB, and the risk in those reporting injectable class A drugs use was much higher, than non-drug users. Reports from 1971, before the HIV pandemic, indicate that the prevalence and incidence of TB among drug users in Harlem, New York, were respectively 9 and 10 times higher than that of its general population^24^, similar to our unadjusted estimates; in that study, tuberculin skin test positivity rates were similar in drug-users and the general population, suggesting that drug use may increase the risk of active TB after infection rather than only *Mycobacterium tuberculosis* infection^24^. Studies from the USA^25^ and the UK^26^ have also found that smear positive pulmonary TB is twice more common in drug-users than other TB forms, and diagnosis delays are more frequent, contributing to more intensive transmission in this population. Recent *TB* infection is in itself a strong TB risk factor, with nearly 5–10% risk of TB in the 2–5 years following infection^27^. HIV co-morbidity and under-nutrition also likely contribute to the higher risk in those using injectable drugs.

We found an independent association between history of homelessness and higher TB risk, after accounting for the other TB risk factors, including tobacco smoking, harmful drug use and alcohol drinking. This can be explained by other TB determinants not explicitly included in our analyses, for instance poorer nutritional status.

The absence of an association between alcohol drinking and TB in our study was consistent with the results of the sole previous UK study in which this was measured^28^. Subjects who reported regularly drinking on average 14 units or more per week, were only about 50% more likely to have TB than non-drinkers, but alcohol was no longer associated with TB after adjusting for other social determinants. In contrast, the pooled relative risk from a previous systematic review suggests nearly three times more TB among heavy drinkers^29^. The difference to our results likely reflects duration of exposure; our study population is younger (23 to 38 years), hence less likely to have been exposed to long-term harmful drinking, whereas studies reporting stronger association included older adults, and/or subjects with recorded diagnosis of alcohol abuse^29^.

Household overcrowding and history of prison stay were both strongly associated with TB, although the associations were weaker after adjusting for other variables, especially tobacco smoking, drug use and homelessness. Overcrowding increases the risk of *TB* exposure and infection^30^, and the higher prevalence of latent tuberculosis infection among prisoners has been noted in several surveys^31^.

Our results are relatively conservative. We included social factors and behaviours perceived as undesirable and typically under-reported; therefore, the associations are more likely to be underestimates. Reasonable efforts were made to include all eligible cases and comparable controls, including for example, attempts to contact eligible TB cases with known contact details at shelters or temporary accommodations^11^. There are limitations to the data presented, however. The retrospective ascertainment of exposure may have led to some misclassification, although most likely non-differential between cases and controls, again leading to underestimation rather than overestimation of the associations measured. Lower success in enrolling TB cases in the most deprived areas^11^ may have further contributed to underestimating the association between lower SES and TB. Finally, a limitation of using DAGs in our analyses is the requirement of relatively simplistic causal assumptions to describe complex relationships between the various social determinants investigated. For example, drug-users are more likely to go to prison, but prisoners can develop drug addiction during their prison-stay^32^.

Overall, our results provide insights into some pathways through which social deprivation affects the risk of TB in the native population of a low-incidence high-income country. TB rates have relatively stagnated in the UK-born population for nearly a quarter of a century in spite of the scaling up of control efforts in recent years; new threats have also emerged, including multi-drug resistant TB strains. The WHO global TB strategy recognises additional actions to address the underlying social determinants of TB are needed to complement current TB control and prevention tools for elimination^8^. The potential impact of interventions like tobacco cessation programmes and accessible substance misuse services on TB rates, as well as the primary prevention of tobacco, alcohol and drug misuse, should be additional reason to allocate resources, besides their other health benefits. Attributable fraction estimates suggest tobacco smoking cessation in white UK-born young adults may help reduce about a fifth TB cases in the target population, while drug addiction prevention could help avert nearly 15% TB. A UK-based qualitative study had highlighted the need to integrate care across a number of social and health services to address the complex needs of TB patients to achieve better treatment outcome^33^. It is encouraging that the Collaborative TB control strategy for England 2015–2020^34^ which is currently being updated, has given more pre-eminence to addressing social determinants of TB, including for example recommendations on closer collaboration between local authorities and health services to address socio-economic risk factors of TB, dedicated TB services for homeless and those attending substance misuse services, fast-track access to social care and accommodation for homeless for the duration of their treatment, continuity of TB care between prison and the community, and the systematic monitoring of social determinants of TB^34^. This study provides further arguments in support of such integrated approaches to reduce the disease risk.

## Supplementary information

Supplementary material.

## Data Availability

The data presented here are available from the London School of Hygiene and Tropical Medicine institutional online data repository (https://datacompass.lshtm.ac.uk/) and on request from the paper senior author (PM).
