# Supplementary material for: Drug misuse, tobacco smoking, alcohol and other social determinants of tuberculosis in UK-born adults in England: a community-based case-control study
Source: Sci Rep. 2020 Mar 27;10:5639. doi: 10.1038/s41598-020-62667-8 (PMC7101386; doi:10.1038/s41598-020-62667-8)
Supplement: Supplementary file 1 — Supplementary material. [file 41598_2020_62667_MOESM1_ESM.docx]

**Title:**

Drug misuse, tobacco smoking, alcohol and other social determinants of tuberculosis in UK-born adults in England: a community-based case-control study

**Supplemental material**

**Author list:**

Patrick Nguipdop-Djomo*, PhD(1); Laura C. Rodrigues, PhD(1); Peter G. Smith, DSc(1); Ibrahim Abubakar, PhD(2); Punam Mangtani, MD(1)

**Affiliations:**

1. Department of Infectious Disease Epidemiology, Faculty of Epidemiology and Population Health, London School of Hygiene & Tropical Medicine, London, UK
2. Institute of Epidemiology and Health, and Centre for Infectious Disease Epidemiology, Faculty of Population Health Sciences, University College London, London, UK

**Correspondence to**: Patrick Nguipdop Djomo, Department of Infectious Disease Epidemiology, Faculty of Epidemiology and Population Health, London School of Hygiene & Tropical Medicine,London WC1E 7HT, UK [**patrick.nguipdop-djomo@lshtm.ac.uk**](mailto:patrick.nguipdop-djomo@lshtm.ac.uk)

45/662 (6.8%)

4/662 (0.6%)

29/662 (4.4%)

7/662 (1.1%)

129/662 (19.5%)

**Homelessness**

**Class A drug use**

**Prison stay**

25/662 (3.8%)

**Cases**

11/1147 (1.0%)

4/1147 (0.3%)

29/1147 (2.5%)

14/1147 (1.2%)

191/1147 (16.7%)

**Class A drug use**

**Prison stay**

6/1147 (0.5%)

**Homelessness**

**Controls**

38/662 (5.7%)

21/1147 (1.8%)

**Supplementary Figure 1:** Prevalence of reported class A drug use, homelessness and prison in cases and controls

**Supplementary table 1:** Characteristics of subjects with and without missing data, and association to missingness

| **Variable** | **Missing data (% (n=226)** | **Complete data (% (n=1638)** | **Crude OR for missingness(95%CI)** | **p-value** |
| --- | --- | --- | --- | --- |
| **TB status** | | | | |
| Controls | 123 (54%) | 1060 (65%) | - |  |
| Cases | 103 (46%) | 578 (35%) | 1.53 (1.16, 2.03) | 0.003 |
| **Birth cohort** | | | | |
| 1985-1989 | 25 (11%) | 207 (13%) | - |  |
| 1980-1984 | 49 (22%) | 367 (22%) | 1.10 (0.66, 1.84) |  |
| 1975-1979 | 52 (23%) | 427 (26%) | 1.01 (0.61, 1.67) | 0.219 |
| 1970-1974 | 75 (33%) | 422 (26%) | 1.47 (0.91, 2.38) |  |
| 1965-1969 | 25 (11%) | 215 (13%) | 0.96 (0.54, 1.73) |  |
| **Sex** | | | | |
| Female | 118 (52%) | 937 (57%) | - |  |
| Male | 108 (48%) | 701 (43%) | 1.22 (0.93, 1.62) | 0.157 |
| **Education level** | | | | |
| Degree level | 51 (31%) | 628 (38%) | - |  |
| A level, SCE Higher | 25 (15%) | 317 (19%) | 0.97 (0.59, 1.60) |  |
| O level, GCE or GCSE | 59 (36%) | 515 (31%) | 1.41 (0.95, 2.09) | 0.020 |
| None | 29 (18%) | 178 (11%) | 2.00 (1.23, 3.26) |  |
| **Small area-level deprivation** | | | | |
| Least deprived quintile | 34 (15%) | 268 (26%) | - |  |
| 2^nd^ quintile | 29 (13%) | 308 (19%) | 0.74 (0.44, 1.25) |  |
| 3^rd^ quintile | 28 (12%) | 316 (19%) | 0.70 (0.41, 1.18) | <0.001 |
| 4^th^ quintile | 44 (19%) | 323 (20%) | 1.07 (0.67, 1.73) |  |
| Most deprived quintile | 91 (40%) | 423 (26%) | 1.69 (1.11, 2.59) |  |
| **Persons per bedroom (ppb)** | | | | |
| <2 ppb | 184 (89%) | 1507 (92%) | - |  |
| ≥2 ppb | 23 (11%) | 131 (8%) | 1.44 (0.90, 2.30) | 0.142 |
| **BCG vaccination status** | | | | |
| Unvaccinated | 41 (19%) | 277 (17%) | - |  |
| BCG vaccinated | 172 (81%) | 1361 (83%) | 0.85 (0.59, 1.23) | 0.401 |
| **Stay of 3 months or more in High TB-incidence areas (Africa or Asia)** | | | | |
| No | 212 (94%) | 1524 (93%) | - |  |
| Yes | 14 (6%) | 114 (7%) | 0.88 (0.50, 1.57) | 0.666 |
| **Tobacco smoking** | | | | |
| Never smoked | 48 (27%) | 651 (40%) | - |  |
| Past smoker | 24 (13%) | 285 (17%) | 1.14 (0.67, 1.90) |  |
| <10 pack-years | 56 (31%) | 423 (26%) | 1.80 (1.20, 2.69) | <0.001 |
| 10-19.9 pack-years | 34 (19%) | 191 (12%) | 2.41 (1.51, 3.86) |  |
| ≥20 pack-years | 16 (9%) | 88 (5%) | 2.46 (1.34, 4.53) |  |
| **Typical Alcohol consumption** | | | | |
| ≤ 40g (5 units)/week | 76 (50%) | 991 (61%) | - |  |
| 41-112g (5-14 units)/week | 43 (28%) | 429 (26%) | 1.31 (0.88, 1.93) |  |
| >112g (14 units)/week | 32 (21%) | 218 (13%) | 1.91 (1.23, 2.97) | 0.017 |
| **Class B and C drugs misuse** | | | | |
| Never used any | 104 (58%) | 1092 (67%) | - |  |
| Used >10 years ago | 20 (11%) | 178 (11%) | 1.18 (0.71, 1.95) |  |
| Used 1-10 years ago | 24 (13%) | 177 (11%) | 1.42 (0.89, 2.28) | 0.062 |
| Used <1 year ago | 32 (18%) | 191 (12%) | 1.76 (1.15, 2.69) |  |
| **Class A drugs misuse** | | | | |
| Never used | 122 (69%) | 1225 (75%) | - |  |
| used >10 years ago | 15 (8%) | 117 (7%) | 1.29 (0.73, 2.27) |  |
| used ≤10 years ago | 31 (17%) | 228 (14%) | 1.37 (0.90, 2.07) | 0.355 |
| Used injectable class A | 10 (5%) | 68 (4%) | 1.48 (0.74, 2.94) |  |
| **History of homelessness** | | | | |
| Never | 174 (84%) | 1486 (91%) | - |  |
| ≤12 weeks | 19 (9%) | 85 (5%) | 1.91 (1.13, 3.21) | 0.012 |
| >12 weeks | 15 (7%) | 67 (4%) | 1.91 (1.06, 3.42) |  |
| **History of prison stay** | | | | |
| Never been in prison | 187 (91%) | 1538 (94%) | - |  |
| Stayed in prison | 18 (9%) | 100 (6%) | 1.48 (0.88, 2.50) | 0.158 |
